# Reemergence of *Echinococcus granulosus* Infections after 2004 Termination of Control Program in Magallanes Region, Chile

**DOI:** 10.3201/eid3102.240980

**Published:** 2025-02

**Authors:** Cristian A. Alvarez Rojas, Juan Francisco Alvarez

**Affiliations:** Pontificia Universidad Católica de Chile, Santiago, Chile (C.A. Alvarez Rojas); Servicio Agrícola y Ganadero, Magallanes Region, Punta Arenas, Chile (J.F. Alvarez)

**Keywords:** *Echinococcus granulosus*, cystic echinococcosis, echinococcosis, cestodes, tapeworms, control program, emergence, parasites, zoonoses, Magallanes Region, Chile

## Abstract

After termination of a control program in 2004, *Echinococcus granulosus* infections have reemerged in Magallanes Region, Chile. Prevalence in sheep >2 years of age in 2023 resembled levels observed at the start of the program. Resurgence underscores the need for continued surveillance, particularly in younger sheep, to monitor recent transmission trends.

Cystic echinococcosis (CE) is a zoonotic disease caused by the taeniid tapeworm *Echinococcus granulosus* sensu lato (*1*). This neglected disease occurs worldwide in humans and animals. Several control programs for CE have been implemented worldwide; eradication by those programs has been achieved in insular settings, such as Iceland (*2*), New Zealand (*3*), and Tasmania (*4*), only after >40 years of intervention (*5*). Although Tasmania was declared provisionally hydatid disease-free in 1996, cases in nonimported dogs, sheep, and young cattle persist in northern Tasmania (*6*,*7*).

In March 2004, rural dogs from the Magallanes Region in southern Chile were the last animals to be treated with praziquantel under the Magallanes hydatid disease control project, which began in 1979 and lasted 25 years. This program substantially reduced CE prevalence in dogs, sheep, and humans. Before 1979, the Magallanes Region had an alarming percentage of dogs (60%) and sheep (82%) infected with *E*. *granulosus*, and human incidence risk reached 46.8 cases/100,000 persons annually. The control program reversed those figures considerably, reducing them to 0.5% in dogs and 0.7% in sheep by 2004; human incidence dropped to 10.4 cases/100,000 persons (Figure). The program is considered one of the most successful interventions for controlling this parasite in continental settings (*5*,*8*). However, the project did not advance to the consolidation phase, which aimed to target farms having persistent dog infections; funding was redirected to address other diseases. We report a reemergence of *E. granulosus* in the Magallanes Region.

**Figure:**
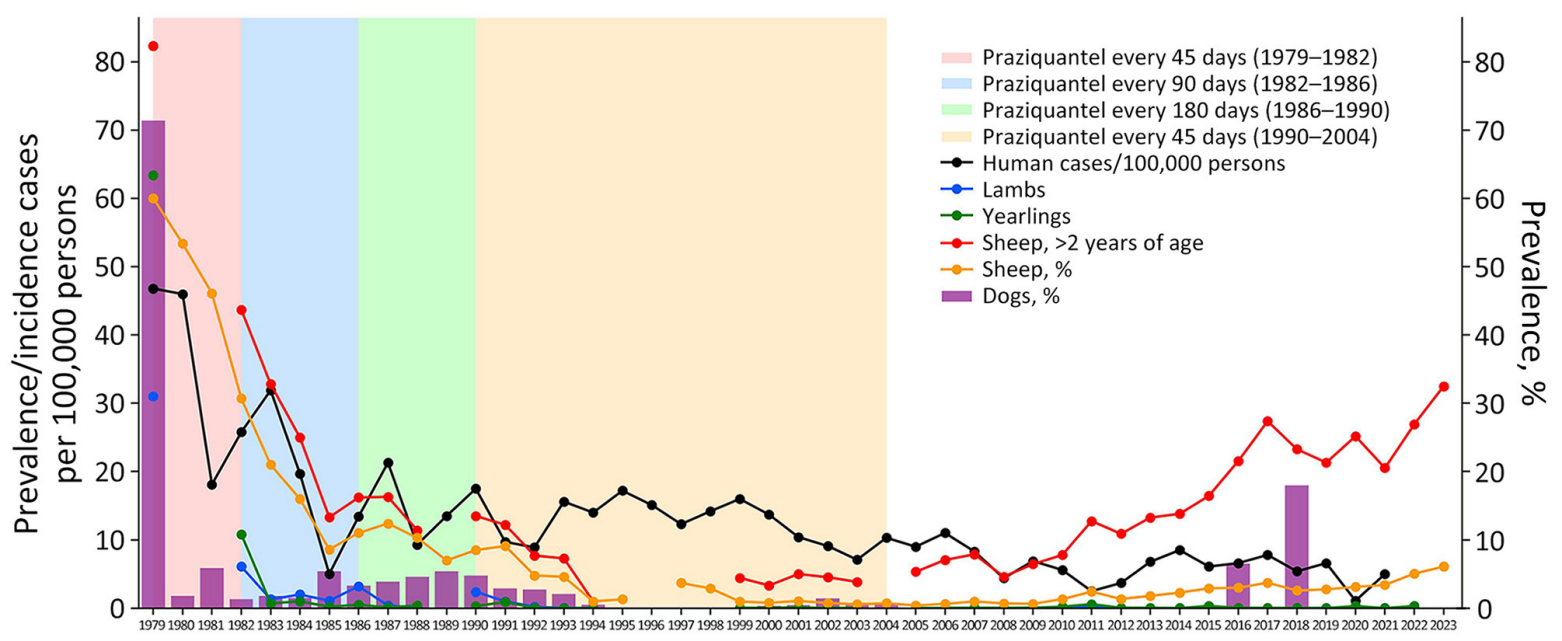
Prevalence of *Echinococcus granulosus* infections after 2004 termination of control program in Magallanes Region, Chile. Prevalence is indicated for humans, sheep, and dogs during 1979–2023. Prevalence of cystic echinococcosis in sheep was calculated according to official condemnation data of viscera in abattoirs; *E. granulosus* infections in dogs was according to arecoline purgation tests until 2004 and human incidence of cystic echinococcosis for the period 1979–2021. Shading indicates praziquantel dosage intervals for treatment of dogs. No data for sheep are presented for 1996 because of the high death rates caused by a large snowfall. Also, in 1998 and 1999, all staff from Servicio Agrícola y Ganadero, Chile, were dedicated to fighting an outbreak of scabies in sheep, and no arecoline purgation tests in dogs were performed.

## The Study

We analyzed data from official reports on viscera condemnation at abattoirs and arecoline purgation tests for dog infections collected by the Servicio Agrícola y Ganadero (SAG) in Chile and obtained human incidence data from hospital records during 1979–2023 (Figure). In 1996, no data were collected because of a severe snowfall, which caused a high number of sheep deaths. In 1998 and 1999, a major scabies outbreak in sheep required the full attention of SAG staff, and, thus, no arecoline purgation tests of dogs were conducted during those years.

After 2004, a steady increase in sheep infections was observed, reaching 6% in 2023. Prevalence of *E. granulosus* infections in sheep >2 years of age increased to 32.5% in 2023, comparable to rates observed in 1983. Linear regression analysis confirmed significant changes in prevalence before and after 2004, indicating the effectiveness of the control program and infection reemergence after its termination (Appendix Figure). Infection data during 1999–2023 might reflect the productive life of sheep, highlighting the need for targeted surveillance and interventions in young sheep that indicate recent infections (Appendix Table).

Human CE incidence in Magallanes Region has fluctuated from 2.5 to 8.5 cases/100,000 inhabitants during 2010–2017. Although some variability is expected at low incidence, the concerning trend is the overall increase in cases during this period, emphasizing the need for continuous monitoring and intervention to address the growing public health threat posed by CE.

We did not explore transmission dynamics; however, sheep and dogs appear to play a central role in domestic transmission. The consistent observation that older sheep represent most infected cases underscores the need for targeted control measures, including vaccination of younger sheep to prevent infection buildup over time. To reinforce the importance of sustained control measures, Chile has piloted vaccination strategies for disease control in sheep (*9*,*10*). Ongoing efforts by SAG in collaboration with regional veterinary authorities aim to reduce the burden of parasitic infections through targeted vaccination campaigns, which could also be a complementary strategy to dog deworming to control CE.

The high percentages of *E. granulosus*–infected older sheep (84%–98%) suggests cumulative exposure over time, and transmission surveillance should also focus on younger animals. A similar trend has been observed in Wales, where older sheep harbored infectious stages of the tapeworm after a control program (*11*). Furthermore, 1 study reported sheep >4 years of age harbored 80% of the protoscolices despite those sheep constituting only 28% of the population at slaughter (*12*). Older sheep play a critical role in CE epidemiology; longer life spans create more opportunities for *E*. *granulosus* egg exposures in pastures. Targeting older sheep for surveillance can help identify infection hotspots and reduce the costs associated with random selection in slaughterhouses. 

Increases in the dog population (>8 million owned dogs) in Chile, particularly in urban and periurban areas, present a considerable challenge for CE control (*13*). Dogs are the definitive hosts for *E. granulosus*, and their feces can contaminate the environment with parasite eggs, promoting transmission to intermediate hosts, such as sheep and humans. The rise in dog populations in Punta Arenas and other parts of Magallanes Region exacerbates this issue, making it imperative to implement effective dog population management strategies, including deworming, vaccination, and public education campaigns. Canine infection data after 2004, although limited, show increased prevalence; 1 study showed spatial clusters in rural areas by using ClusterSeer (BioMedware, https://www.biomedware.com) (*14*). That study analyzed 1,069 environmental dog fecal samples across Magallanes Region by using PCR, revealing substantial *E. granulosus* prevalence, particularly clustered in rural areas (*14*). Another study found a 6.9% prevalence of *E. granulosus* DNA in dog fecal samples from Tierra del Fuego, Chile, indicating continued risk for transmission (*15*).

Fluctuations in human CE incidence indicate a need for improved diagnostic and reporting systems. Enhanced surveillance and reporting mechanisms are essential for accurately assessing the burden of CE and implementing timely and effective control measures. Collaboration among public health authorities, veterinary services, and local communities is crucial for the success of those efforts. Existing technologies have already been integrated into control programs to improve epidemiologic surveillance and population screening. Diagnostic tools, such as coprologic PCR and ELISA, are being used for the detection of *E. granulosus* infections in dogs, offering higher sensitivity and specificity compared with traditional methods. In addition, environmental monitoring of dog fecal samples can identify areas with high levels of contamination. Ultrasound has been applied as a noninvasive method to detect hydatid cysts in older sheep and monitor infection status. Those technologies, combined with regular deworming campaigns, play a crucial role in maintaining effective *E. granulosus* surveillance and ensuring timely interventions.

## Conclusions

The historical context of the Magallanes Region hydatid disease control program provides valuable lessons for current and future control initiatives. The program’s success in reducing *E. granulosus* prevalence underscores the effectiveness of sustained, coordinated efforts in controlling zoonotic diseases. However, the reemergence of infections after ending the program emphasizes the need for sustained funding and political support for such initiatives. Whereas Tierra del Fuego is an island, ongoing control across Magallanes Region and mainland Chile remains crucial to prevent reinfection and manage cross-border transmission risks.

CE remains a considerable public health threat in the Magallanes Region, which has Chile’s largest sheep population, accounting for 57% of the national total. The elevated prevalence of *E. granulosus* in mature sheep reflects their role in sustaining the parasite’s life cycle. Furthermore, increasing dog populations, particularly in urban and periurban areas, raise the risk for environmental contamination with *E. granulosus* eggs and CE transmission within the community. The reemergence of *E. granulosus* after ending the Magallanes Region control program illustrates the critical need for sustained control measures and long-term postelimination surveillance to prevent resurgence. Continued intervention and monitoring of *E. granulosus* infections will be needed to secure lasting public health benefits and similar efforts should be implemented worldwide. 

Appendix Additional information for reemergence of *Echinococcus granulosus* infections after 2004 termination of control program in Magallanes Region, Chile.
