# Supplementary material for: Reemergence of Echinococcus granulosus Infections after 2004 Termination of Control Program in Magallanes Region, Chile
Source: Emerg Infect Dis. 2025 Feb;31(2):373–6. doi: 10.3201/eid3102.240980 (PMC11845154; doi:10.3201/eid3102.240980)
Supplement: Appendix — Additional information for reemergence of Echinococcus granulosus infections after 2004 termination of control program in Magallanes Region, Chile. [file 24-0980-Techapp-s1.pdf]

Article DOI: https://doi.org/10.3201/eid3102.240980

EID cannot ensure accessibility for supplementary materials supplied by authors. Readers who have difficulty accessing supplementary content should contact the authors for assistance.

Reemergence of *Echinococcus granulosus* Infections after 2004 Termination of Control Program in Magallanes Region, Chile

Appendix

Appendix Table. Number of slaughtered and infected sheep during 1999–2023 in study of reemergence of *Echinococcus granulosus* infections after 2004 termination of control program in Magallanes Region, Chile*

Year	No. sheep slaughtered	No. sheep infected	No. sheep >2 years of age (% total infected sheep)
1999	526,000	5,125	5,004 (97.6)
2000	668,912	5,270	5,170 (98.1)
2001	650,363	6,890	6,383 (92.6)
2002	597,603	4,555	4,291 (94.2)
2003	518,272	2,903	2,706 (93.2)
2004	619,256	4,525	ND
2005	557,174	2,125	2,071 (97.4)
2006	597,320	3,726	3,457 (92.7)
2007	641,510	6,294	5,966 (94.7)
2008	671,995	4,697	4,403 (93.7)
2009	637,855	4,042	3,614 (89.4)
2010	673,979	8,975	8,244 (91.8)
2011	653,750	15,828	13,317 (84.1)
2012	561,032	7,588	7,413 (97.7)
2013	495,977	8,982	8,854 (98.5)
2014	582,275	13,227	13,055 (98.7)
2015	537,748	15,683	14,003 (89.2)
2016	486,856	14,588	13,946 (95.6)
2017	525,996	19,688	19,218 (97.6)
2018	532,549	13,781	13,115 (95.1)
2019	518,305	14,342	13,839 (96.4)
2020	519,933	16,213	15,581 (96.1)
2021	491,965	16,811	16,024 (95.3)
2022	512,300	26,329	25,450 (96.6)
2023	514,687	31,727	31,134 (98.1)

*Table shows data for the total number of sheep slaughtered, total number of infected sheep, and number of infected sheep >2 years of age. The percentage of infected older sheep was consistently high, ranging from 77% to 98% annually. ND, no data.

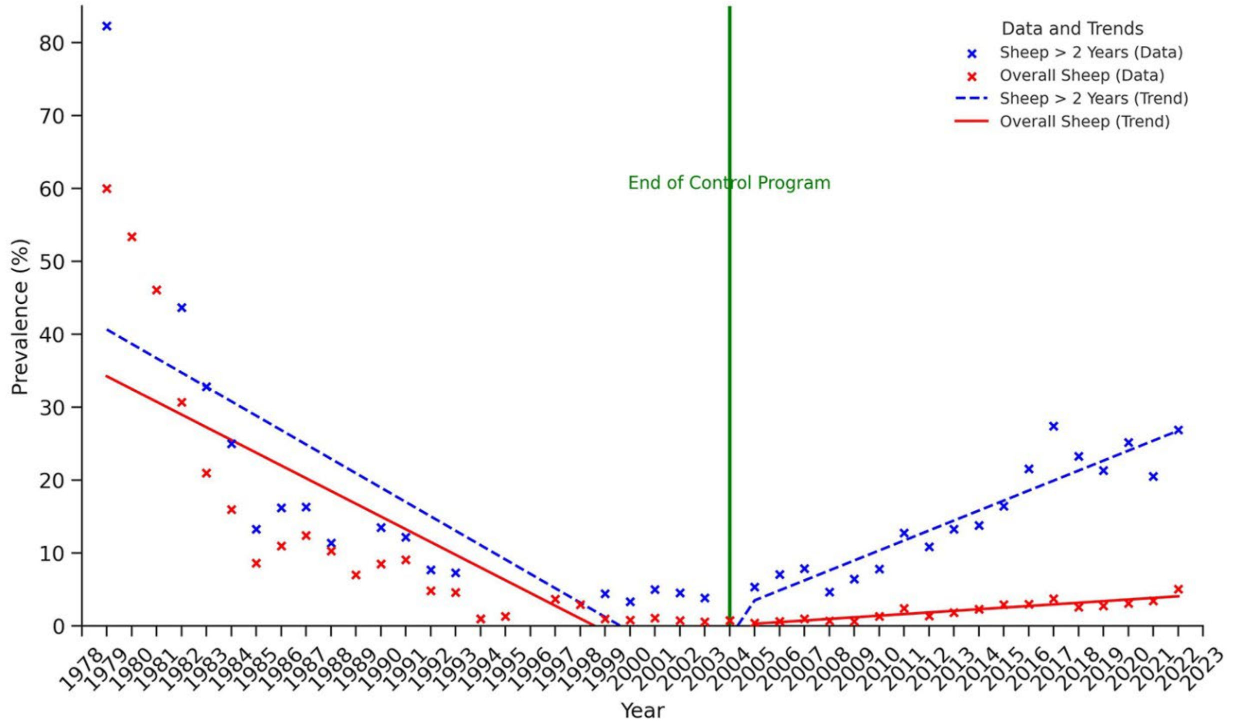

Appendix Figure. Prevalence and trends of *Echinococcus granulosus* infections in sheep after 2004 termination of control program in Magallanes Region, Chile. Data show prevalence of *E. granulosus* infections in sheep >2 years of age compared with all sheep during 1979–2023 before and after the end of the control program in 2004. Blue crosses indicate prevalence of infection in sheep >2 years of age; red crosses indicate prevalence for all sheep. Blue dashed lines indicate the linear regression trends for sheep >2 years of age; a significant decrease in infections was observed before 2004 (slope = −2.420, p = 1.22e-06), and a significant increase was observed after 2004 (slope = 1.397, p = 5.14e-10). Red solid lines indicate the infection trends for all sheep; a significant decrease was observed before 2004 (slope = −1.859, p = 1.04e-06), and a slight but significant increase was observed after 2004 (slope = 0.239, p = 2.60e-08). Green vertical line marks the year 2004, the end of the control program. Linear regression analysis highlights the effect of the control program on reducing sheep prevalence before 2004 and the reemergence of infections after the program ended.
